# Global gene expression analyses of bystander and alpha particle irradiated normal human lung fibroblasts: Synchronous and differential responses

**DOI:** 10.1186/1755-8794-1-63

**Published:** 2008-12-24

**Authors:** Shanaz A Ghandhi, Benjamin Yaghoubian, Sally A Amundson

**Affiliations:** 1 Center for Radiological Research, Columbia University Medical Center, New York, NY 10032, USA

## Abstract

**Background:**

The existence of a radiation bystander effect, in which non-irradiated cells respond to signals from irradiated cells, is now well established. It raises concerns for the interpretation of risks arising from exposure to low doses of ionizing radiation. However, the regulatory mechanisms involved in the bystander response have not been well elucidated. To provide insight into the signaling pathways responding in bystanders, we have measured global gene expression four hours after bystander and direct alpha particle exposure of primary human lung fibroblasts.

**Results:**

Although common p53-regulated radiation response genes like *CDKN1A *were expressed at elevated levels in the directly exposed cultures, they showed little or no change in the bystanders. In contrast, genes regulated by NFκB, such as *PTGS2 *(cyclooxygenase-2), *IL8 *and *BCL2A1*, responded nearly identically in bystander and irradiated cells. This trend was substantiated by gene ontology and pathway analyses of the microarray data, which suggest that bystander cells mount a full NFκB response, but a muted or partial p53 response. In time-course analyses, quantitative real-time PCR measurements of *CDKN1A *showed the expected 4-hour peak of expression in irradiated but not bystander cells. In contrast, *PTGS2, IL8 *and *BCL2A1 *responded with two waves of expression in both bystander and directly irradiated cells, one peaking at half an hour and the other between four and six hours after irradiation.

**Conclusion:**

Two major transcriptional hubs that regulate the direct response to ionizing radiation are also implicated in regulation of the bystander response, but to dramatically different degrees. While activation of the p53 response pathway is minimal in bystander cells, the NFκB response is virtually identical in irradiated and bystander cells. This alteration in the balance of signaling is likely to lead to different outcomes in irradiated cells and their bystanders, perhaps leading to greater survival of bystanders and increased risk from any long-term damage they have sustained.

## Background

The existence of a bystander effect in which cells not exposed to ionizing radiation respond to a stress signal from nearby irradiated cells is now well established. The recent report of tumor induction in an *in vivo *mouse bystander model [1] demonstrates that bystander responses may affect health outcomes. However, it is still not well understood how this indirect stress response may impact the overall risk from low dose radiation exposures. The sharply increasing use of radio-diagnostic procedures makes these questions especially timely.

Bystander studies have employed many diverse models. In some systems, direct cell-to-cell contact and the presence of gap junctions appear to be required [1,2]. In other experiments, shared medium or the transfer of medium from irradiated to non-irradiated cells is sufficient to transmit an effect [3]. Multiple endpoints have been studied as bystander effects. Most, such as sister chromatid exchanges [4], micronucleus formation [5], chromosome aberrations [6], mutation induction [7], and oncogenic transformation [8] are considered deleterious. However, bystander apoptosis [9] and terminal differentiation [10] also occur, possibly representing tissue protective mechanisms. Like responses to direct irradiation, bystander effects may represent a balance between protective and potentially harmful mechanisms. The factors contributing to this balance are currently unknown.

Although various signaling molecules, including cytokines [11], reactive oxygen species [12], nitric oxide [13], calcium [14], cyclooxygenase-2 (*PTGS2*) [15] and MAP kinases [16] have been implicated in the bystander process, the differential signal transduction pathways regulating the responses to bystander damage are not completely understood. In contrast to the prominent role of p53 in cellular responses to direct ionizing radiation exposure, p53 is not required for expression of the bystander effect [17]. However, it has recently been shown that DNA-PKcs and ATM, a major upstream activator of p53, are required for generation of a bystander signal, but not for response to that signal [4]. Thus, the central cellular radiation damage response pathway does appear to have some involvement in bystander signaling.

Many of the cellular responses to direct ionizing radiation exposure are mediated in part through modulation of gene expression. Although translational and post-translational effects are also important, much can be learned from global gene expression studies that compare transcript levels across the entire genome. Accordingly, gene expression profiling has been used to address many questions in radiation biology, including the prediction of radiation sensitivity in tumor cell lines [18,19] or normal tissue [20] and predicting exposure dose for biodosimetry [21]. More sophisticated network analyses of transcriptomic data are also starting to provide insight into signaling pathways and key transcription factors involved in radiation responses [19,22]. Such an approach is well suited to the study of signaling mechanisms involved in cellular crosstalk and bystander responses.

Microarrays have also been used in some bystander studies. Screening of small numbers of genes on membrane-based arrays identified *GJA1 *(connexin-43) [2] and *PTGS2 *(cyclooxygenase-2) [15] as genes expressed at higher levels in bystanders. Whole transcriptome studies have reported differentially expressed genes after medium transfer from irradiated normal human diploid lung fibroblasts [23] and in normal human fibroblasts exposed to a small number of carbon ions targeted to defined sites in the culture [24].

In the present study, we have measured global gene expression in directly irradiated and bystander IMR-90 normal human lung fibroblasts at four hours after exposure to 0.5 Gy alpha particles. We have also monitored micronucleus formation in tandem with gene expression as a physiological indicator of bystander response in each experiment. We used an exposure system that shields half the cells on each dish, allowing both direct cell-to-cell communication and shared medium. Separation of the shielded and exposed sections of the culture allowed us to analyze responding bystanders and the directly irradiated cells generating the signal within the same experiment. We used quantitative real-time PCR to confirm differential expression of 37 genes, and describe a previously un-reported biphasic response of NFκB regulated genes, which is highly synchronous in irradiated and bystander cells. Pathway analysis of our microarray results revealed transcriptional networks centered on p53 and NFκB in directly irradiated cells. In bystander cells, the response of the p53 node was selectively abrogated. In contrast, the response of the NFκB node was nearly identical in bystanders and irradiated cells. Matrix metalloproteinase genes were also coordinately up-regulated in bystanders, indicating a possible tissue remodeling response in bystander cells. Our findings suggest that in fibroblasts, the bystander response centers on stress signaling and cytokines, rather than on classic radiation responses like proliferation, cell death and cell cycle checkpoints. Non-hit cells in proximity to irradiated cells may therefore be involved in regulation of cell and tissue defense in preference to cell fate decisions.

## Methods

### Cell culture, irradiation and RNA isolation

Early passage (population doubling < 35) IMR-90 human lung fibroblasts (Coriell Cell Repository, Camden, NJ) were sub-cultured in Dulbecco's modified Eagle's medium (Invitrogen, Carlsbad, CA) and Ham's F10 medium in a 1:1 mixture plus 15% fetal bovine serum. Mylar-bottomed culture dishes were prepared as described previously [15]. An inner dish with a base of 38-micron-thick Mylar strips was inserted into a larger dish with a 6-micron Mylar base. The 38-micron Mylar completely shields the alpha particles so that only cells on the thinner Mylar areas of the dish were directly irradiated. Cells seeded in these dishes formed a contiguous layer. Cells were exposed to 0 (sham irradiated) or 0.5 Gy ^4^He ions (125 keV/μm) as simulated alpha particles using the track segment irradiation facility of the 5.5-MV Singletron accelerator at the Radiological Research Accelerator Facility of Columbia University. Four independent experiments were conducted.

Directly irradiated (outer dish) and bystander (inner dish) cells were separated at specified times after irradiation and RNA was isolated using Ribopure (Applied Biosytems, Foster City, CA). RNA concentration was measured using a NanoDrop-1000 spectrophotometer (Thermo Scientific, Waltham, MA) and RNA quality was monitored with the Agilent 2100 Bioanalyzer (Agilent Technologies, Santa Clara, CA). All RNA samples had RNA integrity numbers > 9.0 [25] and 260 nm/280 nm absorbance ratios > 2.

### Binucleate Micronucleus assay

Cells were incubated for 24 hours after irradiation in the original dishes, then separated, trypsinized, counted and seeded into chamber well slides with 1μg/mL cytochalasin B (Sigma-Aldrich, St. Louis, MO). Cells were fixed at 72 hours after irradiation and stained with 5% Giemsa (Invitrogen). Samples were blinded, and 500 binucleate cells in each were scored for the presence of micronuclei using established criteria [26].

### Microarray Hybridization and Analysis

Cyanine-3 (Cy3) labeled cRNA was prepared from 0.3 μg RNA using the One-Color Low RNA Input Linear Amplification PLUS kit (Agilent). Dye incorporation and cRNA yield were monitored with the NanoDrop ND-1000 Spectrophotometer. 1.5 μg of cRNA (> 9 pmol Cy3 per μg cRNA) was fragmented, hybridized to Agilent Whole Human Genome Oligo Microarrays (G4112F) using the Gene Expression Hybridization Kit, and washed following Agilent's recommendations. Slides were scanned with the Agilent DNA Microarray Scanner (G2505B) and default parameters of Feature Extraction Software 9.1 (Agilent) were used for image analysis, data extraction, background correction, and flagging of non-uniform features.

Background corrected intensities were log2 transformed and median-normalized in BRB-Array Tools, Version 3.7.0 [27]. Non-uniform outliers or features not significantly above background intensity in 20% or more of the hybridizations were filtered out, leaving 25800 features. A further filter requiring a minimum 1.5-fold change in at least 20% of the hybridizations was then applied yielding a final set of 7793 features that were used for subsequent analyses. The microarray data is available through the Gene Expression Omnibus database using accession number GSE12435.

BRB-Array Tools was used to identify genes that were differentially expressed in directly and bystander irradiated cells using a random-variance paired t-test, an improvement over the standard t-test that permits sharing information among genes about within-class variation without assuming that all genes have the same variance [28]. The test compares the differences in mean log-intensities between classes relative to the expected variation in mean differences computed from the independent samples. Genes with p-values less than 0.005 were considered statistically significant. The false discovery rate (FDR) was also estimated for each gene [29] to control for false positives.

### Quantitative Real-Time PCR (qRT-PCR)

The High-Capacity cDNA Archive Kit (Applied Biosystems) was used to prepare cDNA from total RNA. A custom low-density TaqMan array was designed with validated assays and obtained from Applied Biosystems (Table 1). For gene validation studies, 100 ng cDNA was used as input for low-density arrays. qRT-PCR reactions were performed with the ABI 7900 Real Time PCR System using Universal PCR Master Mix (Applied Biosystems) with initial activation at 50°C for 120 seconds and 94.5°C for 10 minutes followed by 40 cycles of 97°C for 30 seconds and 59.7°C for 60 seconds. Individual assays (Table 2) were designed with the aid of Genscript real time PCR design software (VWR, West Chester, PA) and synthesized by Operon Biotech, Inc. (Huntsville, AL) with 6-carboxyfluorescein (FAM) at the 5' end and FAM-BHQ1 quencher at the 3' end. To optimize conditions for each gene a standard curve for input cDNA was generated using a range of 6 concentrations starting from 1 μg of cDNA. The efficiency of the primer probe sets was determined and the highest efficiency set was chosen for quantification. Input cDNA was set at 10 ng for all samples and genes, and qRT-PCR reactions were performed with the ABI 7300 Real Time PCR System using Universal PCR Master Mix from Applied Biosystems. All samples were run in duplicate reactions.

**Table 1:** Real time PCR assays used on Low-density arrays

**Number**	**Assay ID^a^**	**Gene Symbol**
1	Hs01076359_m1	*CLDN1*
2	Hs01066938_m1	*MDM2*
3	Hs01055329_m1	*GDNF*
4	Hs00999632_g1	*POU5F1*
5	Hs03044953_m1	*DDB2*
6	Hs99999173_m1	*GADD45A*
7	Hs99999152_m1	*ICAM1*
8	Hs99999034_m1	*IL8*
9	Hs99999032_m1	*IL6*
10	Hs99999142_m1	*CDKN1A*
11	Hs99999029_m1	*IL1B*
12	Hs99999028_m1	*IL1A*
13	Hs99999905_m1	*GAPDH*
14	Hs99999904_m1	*PPIA*
15	Hs99999903_m1	*ACTB*
16	Hs00899658_m1	*MMP1*
17	Hs00745167_sH	*MT1X*
18	Hs00748445_s1	*GJA1*
19	Hs00824723_m1	*UBC*
20	Hs00823168_g1	*MT1H*
21	Hs00696862_m1	*PCNA*
22	Hs00384082_m1	*DNAJC4*
23	Hs00358879_m1	*DUSP2*
24	Hs00364485_m1	*CARD9*
25	Hs00369211_m1	*IL33*
26	Hs00231069_m1	*ATF3*
27	Hs00234032_m1	*SERPINB2*
28	Hs00236966_m1	*CXCL2*
29	Hs00234712_m1	*TNFAIP3*
30	Hs00244586_m1	*FDXR*
31	Hs00195584_m1	*S100P*
32	Hs00183740_m1	*DKK1*
33	Hs00187845_m1	*BCL2A1*
34	Hs00167309_m1	*SOD2*
35	Hs00171132_m1	*GDF15*
36	Hs00171061_m1	*CXCL3*
37	Hs00171455_m1	*LIF*
38	Hs00171085_m1	*CXCL5*
39	Hs00163653_m1	*FAS*
40	Hs00165078_m1	*LAMB3*
41	Hs00153133_m1	*PTGS2*
42	Hs00154192_m1	*BMP2*
43	Hs00158127_m1	*ITGA2*
44	Hs01114093_m1	*KYNU*
45	Hs00968305_m1	*MMP3*
46	Hs00960934_m1	*FGF2*
47	Hs00955889_m1	*GJB2*

^a^Assay ID: numbers are from the validated assay database at ABI

**Table 2:** Real time PCR sequences used in individual assays

**Gene name**	**Primer/probe**	**Primer sequence**
*CDKN1A*^a^	forward	5' CTG GAG ACT CTC AGG GTC GAA
	reverse	5' CGG CGT TTG GAG TGG TAG AA
	probe	5' TCA TGC TGG TCT GCC GCC GT

*IL8*	forward	5' AAGACATACTCCAAACCTTTCCA
	reverse	5' CCAGACAGAGCTCTCTTCCA
	probe	5' TGGACCACACTGCGCCAACA

*PTGS2*	forward	5' AAGACATACTCCAAACCTTTCCA
	reverse	5' CCAGACAGAGCTCTCTTCCA
	probe	TGGACCACACTGCGCCAACA

*BCL2A1*	forward	5' TTGGATATATTTACAGGCTGGCT
	reverse	5'GACCTGATCCAGGTTGTGG
	probe	5' CAGGACTATCTGCAGTGCGTCCTACAG

^a^Previously designed sequence reported in [30].

Relative fold-inductions were calculated by the ΔΔC_T _method as previously used [30] and with SDS version 3 software (Applied Biosystems). We measured 7 housekeeping genes on the low-density arrays and applied Genorm [31] to determine the most appropriate genes for normalizing the results. The low-density array data was normalized to the geometric mean of *PPIA *and *UBC*. The individual gene assays in the time-course study were normalized to *ACTB*.

### Gene ontology and pathway analysis

The genes responding significantly (FDR < 10%) to either direct alpha particle or bystander irradiation were imported into PANTHER [32] and the number of genes in each functional classification category was compared against the number of genes from the NCBI human genome in that category. The binomial test was used to statistically determine over-representation of PANTHER classification categories [33]. Bonferroni corrected p-values less than 0.05 were considered significant.

The sets of genes significantly responding to direct or bystander irradiation (p < 0.005) were also imported into Ingenuity Pathways Analysis (IPA) (Ingenuity^® ^Systems, ) to analyze network interactions between the genes. The imported genes were mapped onto a global molecular network developed from information contained in the Ingenuity Pathways Knowledge Base. Networks of these significant genes were then algorithmically generated based on their connectivity. The biological functions that were most significant to these networks were determined, and Fischer's exact test was used to calculate p-values determining the probability that each biological function assigned to a network was due to chance alone. We also identified the IPA canonical pathways that were most significant within the differentially expressed gene sets. Fischer's exact test was used to calculate a p-value for the probability that the association between the differentially expressed genes and the canonical pathway was explained by chance alone.

## Results

### Micronucleus induction in bystander cells

**Figure 1:**
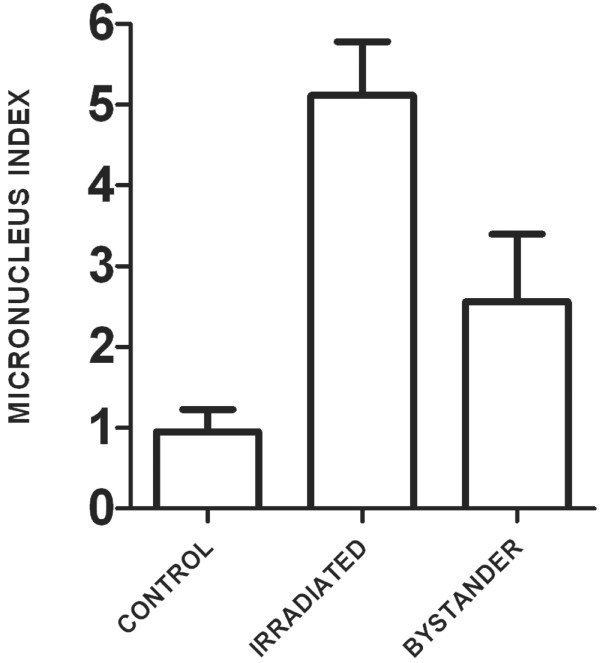
**Micronucleus index of 0.5 Gy alpha particle-irradiated and bystander IMR-90 fibroblasts**. 500 binucleated cells were counted for each condition after 24 hours of co-culture followed by a 48-hour cytochalasin B block. Micronuclei were scored in four independent experiments in parallel with gene expression measurements. Micronucleus index is the percentage of the binucleate cell population with micronuclei. Bars are mean ± standard error of mean.

Cells were irradiated in strip-dishes with 0.5 Gy alpha particles. The fraction of micronucleated binucleate cells was measured as an indicator of DNA damage and genomic instability [26] in parallel with all gene expression experiments. IMR-90 cells responded with a 5- and 3-fold increase in micronucleus frequency in irradiated and bystander cells respectively (Figure 1).

### Gene expression profiles in irradiated and bystander cells

In four independent experiments RNA was extracted 4 hours after exposure of IMR-90 human diploid fibroblasts to 0 or 0.5 Gy alpha particles. In each experiment, RNA from directly-, bystander- or mock-irradiated cells was hybridized to human whole genome microarrays using the Agilent one-color protocol. We used the class comparison feature of BRB-Array Tools [27] to identify genes with significantly different expression levels in IMR-90 fibroblasts 4 hours after direct or bystander exposure to 0.5 Gy alpha particles. In the directly irradiated cells, 300 genes were differentially expressed (p < 0.005; Additional File 1). Of these, 191 had a false discovery rate (FDR) < 10%. In IMR-90 bystander cells 305 genes were differentially expressed (p < 0.005; Additional File 2), 135 of which had a FDR < 10%. 165 genes responded to both direct and bystander irradiation, 73 with FDR < 10% in both conditions (Additional File 3).

### Gene ontology analysis

We analyzed the differentially expressed gene lists from our microarray studies for enrichment of gene groups from the PANTHER database [33], which uses protein sequence information as well as gene families to assign a gene to an ontology group. In directly irradiated cells the most significant gene groups represented pathways including apoptosis and p53 signaling, whereas inflammation and chemokine-cytokine signaling predominated in bystander cells (Table 3). Among biological processes, immunity and defense, signal transduction, and the NFκB cascade were enriched in both conditions. Molecular functions such as signaling molecules, cytokines and chemokines were also enriched in both conditions.

**Table 3:** Gene ontology analysis using PANTHER

**Pathways**	**p-value^a ^Bystander**	**p-value Irradiated**
Inflammation mediated by chemokine and cytokine signaling pathway	6.44 × 10^-4^	NS^b^
Apoptosis signaling pathway	3.29 × 10^-3^	3.46 × 10^-4^
Plasminogen activating cascade	5.92 × 10^-3^	5.61 × 10^-3^
Angiogenesis	2.90 × 10^-2^	NS
Toll receptor signaling pathway	6.46 × 10^-2^	NS
TGF-beta signaling pathway	6.51 × 10-2	NS
p53 pathway	NS	3.87 × 10^-3^
		
**Biological Process**		

Immunity and defense	3.42 × 10^-10^	2.30 × 10^-5^
Signal transduction	1.54 × 10^-9^	5.41 × 10^-6^
Cell proliferation and differentiation	8.99 × 10^-9^	5.74 × 10^-6^
Ligand-mediated signaling	1.72 × 10^-7^	6.83 × 10^-4^
Intracellular signaling cascade	1.29 × 10^-6^	2.43 × 10^-3^
NF-kappaB cascade	2.89 × 10^-6^	4.05 × 10^-5^
Cell communication	7.67 × 10^-6^	NS
Granulocyte-mediated immunity	1.51 × 10^-5^	1.36 × 10^-5^
Cell surface receptor mediated signal transduction	2.40 × 10^-5^	1.68 × 10^-5^
Apoptosis	2.73 × 10^-5^	2.23 × 10^-5^
Inhibition of apoptosis	2.81 × 10^-4^	2.51 × 10^-4^
Macrophage-mediated immunity	5.77 × 10^-4^	4.14 × 10^-3^
Mesoderm development	8.92 × 10^-4^	NS
Cytokine and chemokine mediated signaling pathway	2.58 × 10^-3^	3.76 × 10^-4^
Cell cycle control	NS	4.60 × 10^-4^
Developmental processes	NS	1.65 × 10^-3^
		
**Molecular Function**		

Signaling molecule	6.54 × 10^-11^	2.29 × 10^-6^
Cytokine	3.22 × 10^-5^	3.32 × 10^-4^
Chemokine	8.39 × 10^-5^	1.68 × 10^-2^

^a ^p-values are Bonferroni-corrected

^b ^NS: not statistically significant (p > 0.05).

### Pathway analyses

We next used Ingenuity Pathways Analysis (IPA) to perform pathway analysis of the differentially expressed gene sets. The top interacting networks of radiation responsive genes were significantly enriched for functions of cell death, connective and skeletal development and function (p = 10^-48^), inflammatory and immunological disease, cell-to-cell signaling and function (p = 10^-45^) and cancer, cell death and tumor morphology (p = 10^-29^). The top bystander networks were significantly enriched for connective tissue disorder and inflammatory and immunological disease (p = 10^-48^), connective and skeletal development and function and cellular development (p = 10^-35^) and tissue and cellular growth and proliferation (p = 10^-35^).

**Figure 2:**
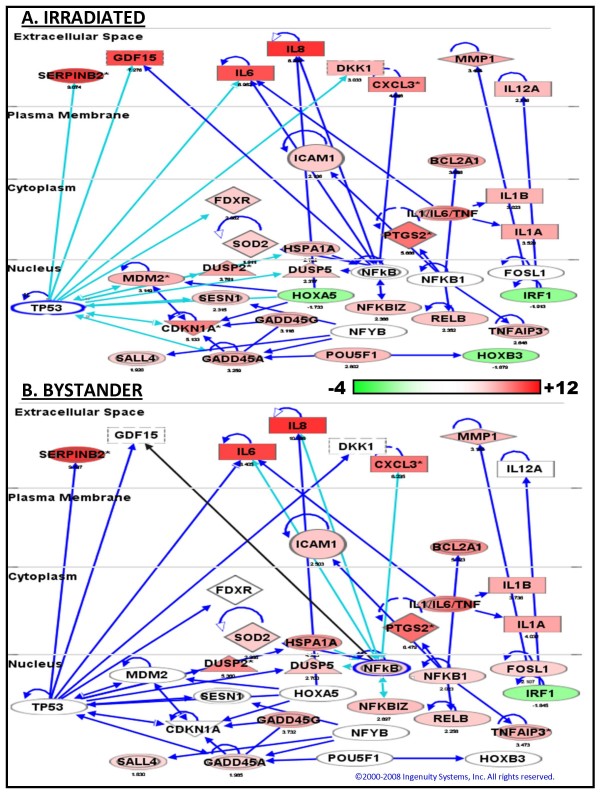
**Network analysis and comparison of gene expression patterns**. Ingenuity Pathways analysis (IPA) was used to generate the network, which has been overlaid with relative gene expression levels of directly irradiated cells (a) and their non-irradiated bystanders (b). Nodes representing gene products are displayed by cellular localization (extracellular space, plasma membrane, cytoplasm or nucleus). The intensity of each node's color indicates the expression level (scale bar) relative to untreated controls. Up-regulated genes are red and down-regulated genes green. Edges (lines and arrows between nodes) represent direct interactions between molecules as supported by information in the Ingenuity knowledge base. Light blue edges highlight direct interactions with p53 (panel a) and NFkB (panel b). The shape of a node represents the functional class of the gene product; rectangles with solid lines for cytokines, rectangles with dotted lines for growth factors, triangles for phosphatases, concentric circles for groups or complexes, diamonds for enzymes, and ovals for transcriptional regulators or modulators.

The top scoring canonical pathway in directly irradiated samples was the p53 signaling pathway (p = 7 × 10^-8^). In bystander samples it was the NFκB pathway (p = 4 × 10^-8^), which was also significant in the directly irradiated samples (p = 4.4 × 10^-3^). In order to visualize interactions that might reveal regulatory hubs we used the genes from the top scoring direct irradiation networks to generate a merged network (Figure 2). Use of the radiation data to generate this network allowed visualization of genes common to the bystander and direct radiation responses, as well as the p53 responses that were not seen in the bystanders. The resulting network has been overlaid with the mean gene expression ratios from the directly irradiated (Figure 2a) and bystander (Figure 2b) experiments. Highly connected hubs such as NFκB in both networks, and p53 in the radiation response network are implicated in regulation of the gene expression response.

### Gene expression levels by real-time PCR

To confirm the microarray results, we measured the expression levels of individual genes using a custom low-density TaqMan PCR array. We selected genes participating in our network (Figure 2) that were significant by microarray analysis in both direct and bystander irradiation, such as cytokine-chemokine signaling related genes including *PTGS2*, *IL6, IL8*, *CXCL2 *and *CXCL3*. Genes responding to direct irradiation with little or no response in the bystanders were also selected. These included p53-regulated genes such as *MDM2, CDKN1A *(p21/WAF1), *GADD45A *and *FDXR*. Quantitative real-time PCR (qRT-PCR) was performed on all samples used for microarray hybridization. The pattern of relative gene expression measured by qRT-PCR agreed with the microarray results (Table 4).

**Table 4:** Comparison of relative gene expression by microarray and qRT-PCR

		**Irradiated**		**Bystander**	
**Gene**	**Description**	**Array^a^**	**qRT-PCR^b^**	**Array**	**qRT-PCR**
*CDKN1A*	cyclin dependent kinase inhibitor, p21	**5.7**	**6.7**	**1.3**	**1.4**
*FDXR*	Ferredoxin reductase	**2.2**	**1.9**	**1.1**	**0.8**
*MDM2*	Human mouse double minute, p53 binding	**2.8**	**3.7**	**1.3**	**1.7**
*GADD45A*	growth arrest & DNA damage inducible	**3.5**	**7.2**	**2.1**	**3.5**
*GDNF*	Glial cell line-derived neurotrophic factor precursor	**5.1**	**8.8**	**1.7**	**3.6**
*DDB2*	damage-specific DNA binding protein 2	**2.2**	**1.9**	**1.1**	**0.9**
*ATF3*	activating transcription factor 3	**1.5**	**2.2**	**0.9**	**1.1**
*FAS*	TNF receptor superfamily, member 6	**2.3**	**3.1**	**1.3**	**1.5**
*GDF15*	growth differentiation factor 15	**8.6**	**9.6**	**3.6**	**3.2**
*IL6*	interleukin 6	**11.2**	**12.7**	**17.1**	**14.7**
*IL8*	interleukin 8	**12.3**	**20.1**	**17.9**	**27.4**
*IL1B*	interleukin 1 beta	**3.6**	**3.4**	**4.4**	**3.9**
*IL1A*	interleukin 1 alpha	**4**	**2.7**	**4.3**	**3.2**
*IL33*	interleukin 33	**6**	**7.8**	**5.4**	**6.7**
*PTGS2*	cyclooxygenase-2	**6**	**6.2**	**6.4**	**6.8**
*CXCL2*	Chemokine CXC ligand 2	**4.1**	**5.9**	**5.2**	**7**
*CXCL3*	Chemokine CXC ligand 3	**6**	**6.9**	**8.9**	**7.9**
*CXCL5*	Chemokine CXC ligand 5	**4.8**	**4.7**	**6**	**5.5**
*BCL2A1*	BCL2 related protein1	**5**	**5**	**7.4**	**5.8**
*LIF*	Leukemia inhibitory factor	**3.1**	**3.5**	**3.7**	**3.7**
*FGF2*	fibroblast growth factor 2	**3.2**	**3.9**	**2.7**	**3.5**
*POU5F1*	POU domain, class 5, transcription factor 1	**3.2**	**2.1**	**1.5**	**1.5**
*ICAM1*	intercellular adhesion molecule 1 (CD54)	**2.3**	**2.5**	**2.6**	**2.7**
*MMP1*	matrix metallopeptidase 1	**2.8**	**3.6**	**3.5**	**3.4**
*MMP3*	matrix metallopeptidase 3	**5.2**	**5.5**	**4.2**	**4.7**
*MT1X*	metallothionein 1X	**4.6**	**13.1**	**4.7**	**17.4**
*MT1H*	metallothionein 1H	**4.6**	**2.8**	**4.7**	**2.5**
*TNFAIP3*	tumor necrosis factor, alpha-induced prot. 3	**2.7**	**3**	**3.4**	**3.4**
*SOD2*	superoxide dismutase 2	**2**	**1.9**	**2.3**	**2.6**
*SERPINB2*	Plasminogen activator inhibitor 2 precursor	**11.4**	**13.9**	**12.6**	**13.3**
*LAMB3*	laminin, beta 3	**2.6**	**2.4**	**3.1**	**2.8**
*KYNU*	kynureninase	**3.1**	**3.3**	**3.6**	**3.9**
*GJA1*	gap junction protein, alpha 1, 43 kDa (connexin 43)	**1.8**	**2.3**	**1.6**	**1.6**
*GJB2*	gap junction protein, beta 2, 26 kDa (connexin 26)	**1.9**	**1.9**	**2.4**	**1.6**
*DUSP2*	dual specificity phosphatase 2	**4.5**	**8.8**	**7.9**	**10.4**
*CARD9*	caspase recruitment domain fam., member 9	**0.7**	**0.6**	**0.7**	**0.5**
*DKK1*	dickkopf homolog 1	**3.2**	**3.1**	**2.4**	**2.7**

^a ^Mean of treated to control ratios measured by microarray in 4 independent experiments.

^b ^Mean of qRT-PCR measurements of the same RNA used in microarray analyses.

### Time course analysis of selected genes

**Figure 3:**
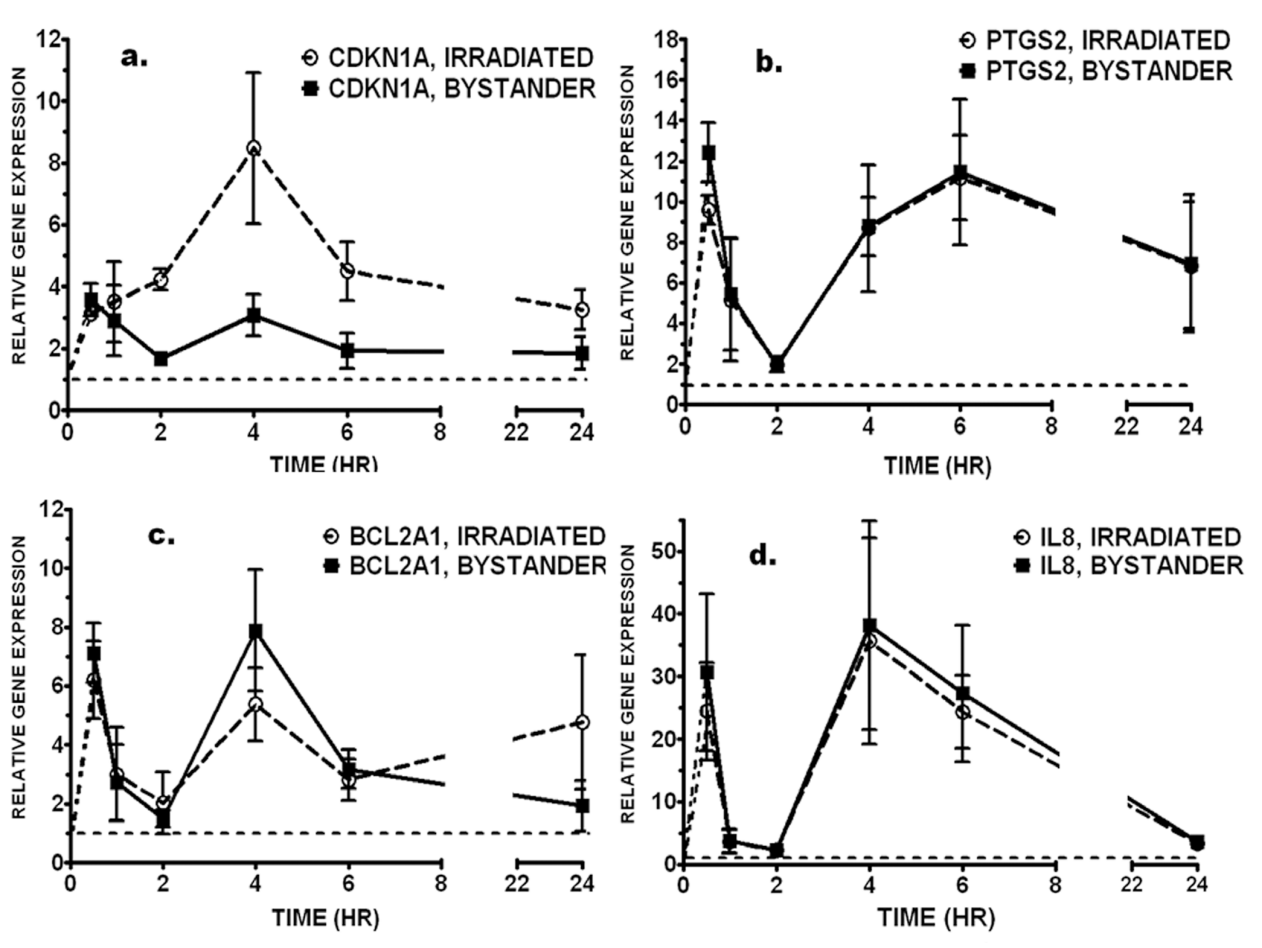
**Time course of gene expression after direct and bystander irradiation**. Quantitative real-time RT-PCR was used to monitor expression of A) *CDKN1A*; B) *PTGS2*; C) *BCL2A1 *and D) *IL8 *at 0.5, 1, 2, 4, 6 and 24 hours after direct irradiation (open circles) or bystander exposure (black squares) of IMR-90 cells. Gene expression was normalized to *ACTB *mRNA levels and is relative to expression in time-matched controls (dashed line). Points are the mean and standard error of four independent experiments.

To investigate the possibility that the responses of directly and bystander irradiated cells may differ in their timing, we also collected RNA at 0.5, 1, 2, 4, 6 and 24 hours after irradiation. We used qRT-PCR to measure expression of *CDKN1A*, *IL8, PTGS2 *and *BCL2A1*. The p53 response gene *CDKN1A *showed a gradual increase in expression with a maximum at 4 hours after direct irradiation, consistent with the pattern observed in other cell lines [34]. In contrast, *CDKN1A *mRNA levels in bystander cells reached a maximum at 0.5 hours after irradiation and remained slightly elevated through 24 hours (Figure 3a). The other three genes, *IL8, PTGS2 *and *BCL2A1*, all showed a similar biphasic pattern with a strong early response at 0.5 hours after irradiation followed by a decline to near background levels between 1 and 2 hours, and then a second peak of expression at 4–6 hours after irradiation. In contrast to *CDKN1A*, the response patterns of these three genes were virtually identical in irradiated and bystander cells (Figure 3b–d).

## Discussion

Both direct and bystander exposure of human fibroblasts to alpha particle irradiation increased formation of micronuclei and altered the global gene expression profiles. Micronucleus formation, a measure of unrepaired DNA damage, was monitored in all experiments as a cellular indicator of bystander effects. Both direct and bystander irradiation consistently induced micronuclei (Figure 1). Despite carefully controlling parameters such as cell age, plating density and time on Mylar prior to irradiation, slight differences in parameters such as extent of cell-to-cell contact within the monolayers can still occur between experiments. It was not possible to maintain confluent cells for many days on the Mylar dishes as is often done to standardize fibroblast cultures, since the cells rapidly detach from the Mylar surface under these conditions. Such variations between expriments may have had a somewhat larger effect on gene expression in bystander cells, where the induction of some genes varied more between experiments than in directly irradiated cells. This is reflected in larger error bars for the bystanders at specific time points, as in Figure 3. This may be an effect of the indirect nature of the inducing signal in bystander cells combined with normal inter-experimental variations.

We identified 305 genes responding in the bystander cells, and 300 in the directly irradiated cells. qRT-PCR was used to confirm differential expression of 37 of these genes (Table 4). The microarray analysis overestimated relative changes of two genes by more than 20%, but both were still up-regulated by the qRT-PCR measurement. Nearly a third of the validated genes showed greater fold-changes by qRT-PCR than predicted by the arrays. Such ratio compression is often encountered in microarray experiments, and is thought to be sequence dependent [35,36]. In all, our microarray analysis was well supported by the qRT-PCR results.

There was a broad similarity between the gene expression response to direct alpha particle exposure and previously reported responses of primary fibroblasts [37-39] and other cell types to x- and gamma rays, in that the major functional categories of responding genes were cell cycle regulation, apoptosis, damage response, and signaling pathways. The alpha particle response showed a strong contribution of p53-regulated genes similar to that commonly seen in other cell lines [19,34,37,38] in response to x rays or gamma rays, and consistent with a prior study of gamma ray irradiated IMR-90 fibroblasts, in which the classic DNA damage response genes were found to be the most responsive [39]. Genes such as *GADD45A, BTG2, IL11, MT1G *and *DKK1 *were responsive to both gamma rays [39] and direct alpha particle irradiation in IMR-90. Potential differences in responses due to differences in radiation quality cannot be inferred from comparisons of these studies, however. Differences in cell strains, experimental protocols, radiation dose and the timing of the gene expression assays, all of which vary among published reports, will result in different gene expression patterns, and preclude such direct comparisons.

**Figure 4:**
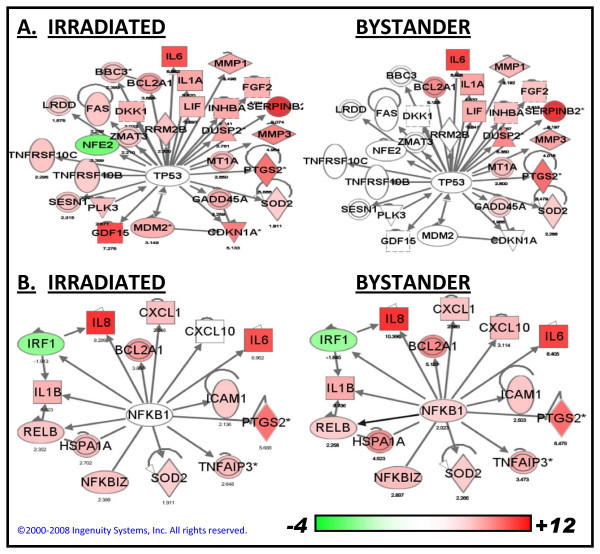
**Responses of genes regulated by p53 and NFκB**. Ingenuity Pathways Analysis was used to extract nodes having direct regulatory interactions with p53 or NFκB from the set of genes significantly responding to radiation. Expression levels are overlaid on the nodes so that up-regulated genes are displayed in red and down-regulated genes in green. The scale bar shows the range of fold-changes. Panel a) p53 regulated genes are overlaid with relative expression levels in irradiated (left) or bystander (right) cells 4 hours after treatment. Just under half the genes changing in irradiated cells respond in bystanders. Panel b) NFκB regulated genes overlaid with relative expression in irradiated (left) and bystander (right). The responses are nearly identical at the 4-hour time point.

Gene ontology analysis indicated a significant (p < 0.004) contribution of the p53 pathway among the genes responding to direct but not bystander irradiation (Table 3). Network analysis (Figure 2) similarly suggests a lesser contribution of p53 to bystander response. Although p53 has been shown not to be required to either generate or respond to a mutagenic bystander signal [17], some activation of p53 would be consistent with prior studies, in which phosphorylation of p53 Ser15 has been reported in bystander cells [40]. Just under half of the p53-regulated genes responding in directly irradiated cells also responded to bystander radiation in our study (Figure 4a), although many showed a smaller magnitude of change than in the directly irradiated cells. These responses could represent regulation by other transcription factors. For instance, about a quarter of the p53 genes responding in the bystander cells are also known targets of NFκB.

Alternately, p53 may be differentially activated in bystander cells. It is known that different stresses and different cellular contexts can result in different patterns of p53-dependent gene expression [34]. Differential regulation of p53 activity is also thought to help explain the switch between the cell protective and cell killing functions of p53 [41,42]. Interestingly, among the p53 genes with apoptosis-related gene ontology annotations, 5 of 11 genes responding to direct irradiation are apoptosis-promoting and 6 of 11 are anti-apoptotic. In contrast, only six of these genes respond to bystander irradiation, including five annotated as anti-apoptotic, and only one annotated as pro-apoptotic. This may suggest activation of a more specialized protective response of the p53 pathway in bystander cells. The balance of pro- and anti-apoptotic signals in a cell is influenced by factors in addition to p53, however. Since apoptosis is not efficiently induced by radiation in primary fibroblasts, the up-regulation of pro-apoptotic genes by direct radiation does not appear to be sufficient to tip the overall balance toward programmed cell death in this cell type.

In contrast to p53, genes regulated by NFκB responded almost identically in irradiated and bystander cells (Figure 4b). Gene ontology analysis indicated that the NFκB cascade was a highly significant process in response to both treatments (Table 3). Activation of NFκB has been demonstrated in bystander cells, and its chemical inhibition has also been shown to reduce mutant fraction in bystander cells [16], consistent with a central role in bystander responses. Related processes of inflammation, immunity and cell signaling were also highly significant in bystander cells (Table 3). The products of many of the genes responding to both direct and bystander irradiation are signaling molecules that are expressed extracellularly (Figure 2), making them attractive candidates for soluble mediators of the bystander effect. Indeed, *IL8*, one of the most robust responders in both irradiated and bystander cells, was one of the first factors implicated in transmission of a bystander signal [43].

In addition to elevation of chemokine and cytokine signaling molecules, PANTHER gene ontology analysis also indicated a significant radiation and bystander response of the plasminogen activating cascade (Table 3). This pathway regulates remodeling of the extracellular matrix (ECM) and is a common marker of metastasis [44] and chronic inflammation [45]. Genes from this pathway that were expressed at higher levels in both irradiated and bystander fibroblasts (Table 4, Additional File 3) included *SERPINB2*, and the matrix metalloproteinase (MMP) genes *MMP1, MMP3 *and *MMP10*, as well as *ICAM1*, a regulator of MMP levels [46]. A previous bystander study also reported up-regulation of *MMP1 *and *MMP3 *in irradiated fibroblasts, although medium transfer did not elicit a response from these genes [23], perhaps indicating a requirement for direct cell-to-cell contact, or possibly even an ECM-mediated effect. *MMP17 *has also been reported as over-expressed in bystander, but not directly irradiated, fibroblasts [24]. Although SERPINB2 is a negative regulator of tissue degradation, the MMPs are positive regulators and would be expected to promote tissue and matrix degradation. These opposing activities may represent a self-limiting activation of ECM remodeling, which represents a novel potential bystander response.

In contrast to results with fibroblasts, *MMP2, MMP3 *and *MMP9 *were reported to be sharply but transiently down-regulated by irradiation of human lens epithelial cells [47], although ICAM1 was up-regulated in these cells [48]. This pattern of responses may be specific to lens epithelium, possibly contributing to radiation cataractogenesis. Bystander effects on the ECM might similarly be expected to be tissue specific.

Because the bystander signal must be generated in the irradiated cells, then transmitted to bystanders, it might be reasonable to expect a delay in the bystander response due to the time required to produce and transmit a signal from the irradiated cells. Indeed, a lag of four hours was recently reported between irradiated and bystander cells for p53 phosphorylation [40]. To check for a possible lag effect on gene expression, we measured the response over time of one gene responding predominantly in the directly irradiated cells (*CDKN1A*) and three genes responding similarly in irradiated and bystander cells (*IL8*, *PTGS2 *and *BCL2A1*). The slight response of *CDKN1A *in bystanders was confirmed, and was not due simply to a difference in timing, as slightly elevated levels of this gene remained steady between 30 minutes and 24 hours after irradiation (Figure 3a).

The expression pattern of the other three genes showed a striking biphasic response, with nearly identical magnitudes of induction in irradiated and bystander cells at all times (Figure 3b–d). This suggests a common regulator activated by the same pathway in both irradiated and bystander cells. A similar biphasic pattern has been reported for *FGF2 *expression, with peaks at 0.5 and 3–4 hours after exposure of human lens epithelial cells to helium ions [49]. This pattern also parallels that reported for NFκB binding in response to TNFα treatment of human T-cells and mouse fibroblasts [50]. These waves of NFκB activity are thought to arise from oscillations between NFκB and its inhibitor IκB. A similar biphasic activation has been reported for receptor tyrosine kinases, which are activated within minutes of radiation exposure [51]. For instance, phosphorylation of ERBB1 peaks at 5–10 minutes after irradiation, returns to baseline levels, then shows a second wave of activation, most likely in response to the release of factors such as TGF-α [52].

We found no lag of gene expression response in bystander cells, even at the earliest time measured (30 minutes). This virtually simultaneous response in bystander and irradiated cells implies a very fast-moving signal. One group, using γ-H2AX foci as a rapid indicator of bystander responses, has been able to time the movement of the bystander signal through a culture of fibroblasts. They reported significant increases in γ-H2AX foci 2.5 millimeters away from the irradiated cells within 2 minutes [53]. Since in our system, no bystander is more than about 1 millimeter away from irradiated cells, this rate of signal transmission would be consistent with our observation of essentially no lag time between responses in irradiated and bystander cells.

## Conclusion

Analysis of directly irradiated normal human fibroblasts and their non-irradiated bystanders has implicated two major regulatory hubs in the global gene expression response. Genes regulated by p53 respond preferentially in directly irradiated cells with only muted responses in bystanders. Conversely, the responses of genes regulated by NFκB are virtually identical in both irradiated and bystander cells. These genes show a distinctive biphasic response suggestive of complex regulatory pathways synchronized throughout the entire culture, in irradiated and bystander cells alike. The greater relative contribution of signaling through NFκB in bystander cells may tip the balance toward survival of these cells, even in the presence of persistent damage, possibly putting bystander cells at increased risk for long-term consequences of radiation damage.

## Competing interests

The authors declare that they have no competing interests.

## Authors' contributions

SAG participated in the design of the study, carried out the microarray analyses and real-time PCR, and contributed to writing the manuscript. BY carried out the micronucleus assays and contributed to tissue culture and RNA isolation. SAA conceived of the study, participated in its design and data analysis, and helped to draft the manuscript. All authors read and approved the final manuscript.

## Pre-publication history

The pre-publication history for this paper can be accessed here:



## Supplementary Material

Additional file 1 **Genes responding 4 hours after 50 cGy direct Alpha particle irradiation.** Genes with significantly altered expression in directly irradiated cells 4 hours after exposure to 50 cGy alpha particle irradiation. Click here for file

Additional file 2 **Genes responding 4 hours after bystander treatment.** Genes with significantly altered expression in bystander cells 4 hours after treatment. Click here for file

Additional file 3 **Genes responding 4 hours after treatment in both directly irradiated and bystander cells.** Genes with significantly altered expression 4 hours after treatment in both directly irradiated and bystander cells. Click here for file
